# The central role of social support in the health of Chinese and Korean American immigrants

**DOI:** 10.1016/j.socscimed.2021.114229

**Published:** 2021-07-14

**Authors:** Brittany N. Morey, Connie Valencia, Hye Won Park, Sunmin Lee

**Affiliations:** a University of California, Irvine, Program in Public Health, Department of Health, Society, & Behavior, 653 E. Peltason Dr., Anteater Instruction and Research Building (AIRB) 2022, Irvine, CA, 92697-3957, USA; b University of California, Irvine, Program in Public Health, Department of Health, Society, & Behavior, 653 E. Peltason Dr., Irvine, CA, 92697-3957, USA; c University of California, Irvine, School of Medicine, Department of Medicine, 1001 Health Sciences Rd., Irvine, CA 92617, USA

**Keywords:** Social support, Self-rated health, Stress, Distress, Mediation, Asian Americans, Immigration, Acculturation

## Abstract

Prior research contends that social support positively influences health by optimizing individuals’ psychological processes such as appraisals, emotions, and sense of control—known as stress-buffering effects. This study tests this theoretical concept by examining whether the association between social support and health can be explained by the psychological processes of perceived stress and distress among Chinese and Korean American immigrants. Furthermore, we examine what predicts social support in this population, with a particular focus on factors related to immigration. Using a total sample of 400 Chinese and Korean American immigrants, we examine the association between social support and self-rated health (SRH), accounting for demographic factors, socioeconomic status, perceived stress, and perceived distress using multivariable logistic regression models. We conducted a mediation analysis using the Karlson, Holm, and Breen (KHB) method to determine whether perceived stress and distress partly explained the association between social support and SRH. Findings showed a strong total effect of higher social support on better SRH. Furthermore, mediation was detected, with perceived stress and distress explaining 42.98% of the total effect of social support on SRH. Multivariable linear regression models revealed that social support among Chinese and Korean American immigrants was associated with marital status, employment, ethnic identity, and acculturative stress. This study highlights the centrality of social support for Chinese and Korean American immigrants, which lowers perceived stress and distress, leading to better overall health. By confirming these stress-buffering effects, our findings suggest that bolstering social support among vulnerable Chinese and Korean American immigrant populations can have a positive effect on health by optimizing stress appraisals.

## 1. Introduction

Social support has been identified as an important social determinant of health. One of the main theoretical mechanisms that links social support to overall health is psychological processes such as appraisals, emotions or moods, and feelings of control—processes also commonly referred to in the literature as stress-buffering effects (Cohen and Wills, 1985; Thoits, 2011; Uchino, 2006). According to this social science theory, social support helps individuals cope with distressing situations in life (Thoits, 2011). The coping assistance that social support provides lessens the psychological consequences of stressful life demands, bolstering the sense of personal control and helping individuals to sustain positive emotions. Therefore, social support indirectly affects health by optimizing individuals’ psychological processes in response to situational challenges in life.

The stress-buffering effects of social support have been demonstrated in relation to health in mainly western and individualistic cultures such as the United States (U.S.) context. Researchers have called for further investigation into whether these mechanisms are applicable to other cultures, especially those that are more collectivistic and value interdependence over independence (Sangalang and Gee, 2012; Thoits, 2011; White et al., 2009). Asian cultures are often considered more collectivistic, and there is some research showing the association between social support and health in countries such as China (Dai et al., 2016; Yang and Jiang, 2020). However, the literature is still emerging on the effects of social support on health among Asian Americans. There is evidence of higher levels of social support being associated with lower levels of psychological distress, depression, and anxiety among foreign-born and U.S.-born Asian Americans (Sangalang and Gee, 2012; Singh et al., 2015). Furthermore, research has shown that social support has direct effects on hypertension and cardiovascular reactivity among Asian American immigrants (Lee et al., 2012; Lu et al., 2019). Yet research has yet to demonstrate the indirect effects of social support on health via psychological processes such as stress reactions, personal control, and emotions among Asian American immigrants.

Previous work examining the stress-buffering effects of social support among Asian Americans have tested for moderation between social support and stress on health (Fang et al., 2020; Lu et al., 2019; Singh et al., 2015). However, the evidence of interactions between social support and stress affecting health is mixed. One study among Asian Americans found that the effect of discrimination on psychological distress was moderated by social support from friends, such that greater friend support lessened the effect of high discrimination on psychological distress (Singh et al., 2015). Furthermore, research among Chinese immigrants suggests that interaction between stressors and social support on depressive symptoms differs by gender, such that high social support lessens the effect of acculturative stress on depressive symptoms only for men, but not for women (Fang et al., 2020). Testing for moderating effects assumes that the effect of stressors varies by social support. However, other work found no significant interactions between social support and perceived stress on hypertension (Lu et al., 2019). It is possible that no interactions were found in this latter case, because the interaction tested is between social support and the psychological stress appraisal (e.g. perceived stress) rather than the experienced stressor (e. g. discrimination, acculturation, etc.) (Uchino, 2006). Additionally, the studies finding significant interactions between social support and stressors examined mental health as the outcome, while the latter study used hypertension as the outcome. According to the stress-buffering theory, social support should lessen the psychological effects of stress in the presence of a stressor, which then protects health (Cohen and Wills, 1985; Uchino, 2006). Therefore, when examining how social support affects health through the psychological process of decreasing individuals’ appraisals of stress, a mediation analysis may be more appropriate than a moderation analysis (Szkody and McKinney, 2020). No study that we are aware of has simultaneously studied the direct effect of social support on health and the indirect effect of social support on overall health through the easing of psychological stress processes among Asian American immigrant groups.

Asian American immigrants have experiences that are unique from their U.S.-born counterparts that may influence levels of social support. For example, Asian American immigrants have the experience of leaving social networks in the country of origin and forging new social networks in the U.S. (Xu and Chi, 2013). Therefore, social support among Asian American immigrants may vary depending on the length of time they have lived in the U.S., with those who lived for a longer amount of time in the U.S. having more time to build social support (Ro, 2014). Furthermore, Asian American immigrants experience different levels of acculturation, independent of time lived in the U.S. (Sullivan and Kashubeck-West, 2015). Some immigrants may maintain a strong identity with their Asian origins, while others may identify more with the American culture that they have chosen to embrace. Still, others may be able to navigate both Asian and American cultures equally. This ethnic identity may affect and be influenced by the types of social networks that people have (Lee and Ciftci, 2014; Umaña-Taylor, 2011). In prior work, Chinese immigrants who identified more strongly with Chinese cultural values reported higher social support (Lee et al., 2012). Social support may also be influenced by the types of conflicts and stressful situations that immigrants uniquely find themselves involved in, such as homesickness, language barriers, legal problems, and nationality discrimination—commonly referred to as acculturative stress (Smart and Smart, 1995; Yakushko et al., 2008). Research points to higher acculturative stress being associated with lower social support (Singh et al., 2015). Moreover, Asian immigrant adults are more likely to be married and to live in intergenerational homes than the U.S. population as a whole; therefore, marital status and household size may also predict greater social support (Pew Research Center, 2013). Social support has additionally been shown to vary by Asian ethnic subgroup, with Chinese and Vietnamese Americans reporting higher levels of social support than Korean Americans (Lu et al., 2019).

This study aimed to examine the stress-buffering hypothesis in a sample of Chinese and Korean American immigrants living in the Baltimore-Washington DC Metropolitan Area. Chinese and Korean Americans are two of the largest Asian groups in this geographic area, making up about 18% and 12% of the total Asian American population, respectively (U.S. Census Bureau, 2021). Previous assessment of Asian Americans in Maryland has noted many health needs among Chinese and Korean communities (Lee et al., 2011). Our study first determined the direct effect of social support on self-rated health (SRH) and the indirect effect of social support on SRH through psychological processes of perceived stress and distress. After determining the association and pathways between social support and health, we explored which factors were associated with greater social support among Chinese and Korean immigrants. Overall, we tested the following hypotheses:

1. Higher level of social support is associated with better SRH among Chinese and Korean American immigrants.
2. Perceived stress and distress partly explain the association between social support and SRH.
3. Social support is associated with factors related to the immigrant experience among Chinese and Korean American immigrants, such as years in the U. S., ethnic identity, and acculturative stress.

## 2. Methods

The data were from the baseline survey of the Screening to Prevent Colorectal Cancer (STOP CRC) Among Chinese and Korean Americans Study—a randomized controlled trial to increase colorectal cancer screening among Chinese and Korean Americans living in the Baltimore-Washington DC Metropolitan Area. The total sample size was 400, which included 200 Chinese and 200 Korean American participants. All participants were foreign-born and recruited from primary care physicians’ clinics. Baseline data were collected between August 2018 and June 2020. Most of data collection (92%) occurred via in-person self-administered questionnaires. However, 8% of data were collected by researcher-led phone survey from March 2020 through June 2020 due to the COVID-19 pandemic. Survey questionnaires were administered in the preferred language of the participant, either in Mandarin, Korean, or English. Study materials were translated by a first bilingual staff member and reviewed by a second bilingual staff member. All 200 Korean participants completed the survey in the Korean language, 198 Chinese participants completed the survey in Mandarin, and two Chinese participants completed the survey in English. Study participants were between the ages of 50 and 75 years old. All participants provided informed consent. The study protocol was approved by the institutional review boards of the University of Maryland and the University of California, Irvine.

### 2.1. Main variables

Health was assessed using SRH, a commonly used measure of overall health and well-being (Jylhä, 2009; Schnittker and Bacak, 2014; Wu et al., 2013). Participants were asked to rate their general health as excellent, very good, good, fair, or poor. Similar to other studies, we dichotomized SRH into “good/very good/excellent” and “fair/poor” (Caetano et al., 2013; Wu et al., 2013).

Social support was measured using an adapted version of the Duke-UNC Functional Social Support Questionnaire, which consists of eight items describing things that other people (family, friends, relatives, co-workers, etc.) could do for the respondent that may be helpful or supportive (Broadhead et al., 1988). The eight items reflect the three main types of social support commonly cited in the literature: emotional (expressions of empathy, love, trust, and caring), instrumental (tangible aid and service), and informational (advice, suggestions, and information) (Heaney and Israel, 2008). Examples of items include: “I have people who care what happens to me,” “I get chances to talk to someone I trust about my personal or family problems,” and “I get help when I am sick in bed.” For each item, response choices were coded on a five-point Likert scale ranging from one (“much less than I would like”) to five (“as much as I would like”). The social support scale was calculated as the mean score across the eight items and ranged from one to five, with higher scores indicating greater social support. The social support scale showed good internal consistency in the total sample (Cronbach’s alpha: 0.92) and was similar among Korean and Chinese American subgroups (Cronbach’s alpha: 0.93 and 0.90, respectively).

Two variables were included as potential mediators of the association between social support and SRH: perceived stress and distress. Perceived stress was measured using a modified version of Cohen’s 14-item Perceived Stress Scale (Cohen et al., 1983), which measures self-reported stress over the past month. This modified scale consists of 10 of the 14 items to measure respondents’ feelings and thoughts, including: “how often have you been upset because of something that happened unexpectedly?” “how often have you felt confident about your ability to handle your personal problems?” and “how often have you been able to control irritations in your life?” Each item was coded on a five-point Likert scale ranging from zero (“never”) to four (“very often”). Items were coded such that higher scores indicated greater frequency of stress. Therefore, six of the 10 items that indicated effective coping with stress were reverse coded such that less frequent coping indicated higher levels of perceived stress. We calculated perceived stress as the sum of responses to all 10 items, with a total possible score ranging from zero to 40 (Cronbach’s alpha: 0.71).

Perceived distress was measured using a single item known as the distress thermometer (Donovan et al., 2014; Roth et al., 1998). Respondents were shown a picture of a thermometer numbered from zero at the bottom (indicating no distress) to 10 at the top (indicating extreme distress). Respondents were asked to circle the number zero to 10 that best described how much distress they have been experiencing in the past week including the day of interview. For participants who completed the phone survey, the survey with all questions were sent to participants in advance, so that the participants could view the survey (including the figure of a thermometer) as they responded by phone. The researcher asked the participant to verbally provide the number zero to 10 that described their level of distress. Perceived distress was treated as a continuous scale with higher scores indicating greater distress.

### 2.2. Covariates

Covariates included age (continuous), gender (male or female), marital status (married/cohabitating or not currently married), and Asian subgroup (Korean or Chinese). These variables may be associated with both social support and SRH simultaneously (Caetano et al., 2013; Donnelly et al., 2019; Lu et al., 2019). In models predicting SRH on social support, we included these variables to rule out confounding due to these factors. We also sought to examine how social support is influenced by these social determinants.

Socioeconomic status has in many previous studies been shown to be a fundamental cause of disease, shaping morbidity and mortality such that higher socioeconomic status is commonly associated with better overall health (Phelan et al., 2010; Stringhini et al., 2012). Furthermore, socioeconomic status may influence social support, and if so, may confound the associations between social support and SRH. Measures of socioeconomic status in this study included education, income, and employment. Education was categorized as less than high school, high school graduate or general education degree (GED), some college or vocational school, college graduate, or attended graduate or professional school. We categorized income as: less than $20,000, $20, 000-$39,000, $40,000-$59,000, $60,000-$79,000, $80,000-$99,000, or $100,000 or more. Employment was three categories: full time, part time, or not employed. Health insurance status may also influence SRH, such that people with more access to health care have better SRH (Prus, 2011). People with greater social support may seek out health care to a greater extent (Thoits, 2011). We included health insurance status as a covariate, which we categorized as having private insurance, Medicare or Medicaid, or no insurance.

In this study, we further examined which factors may influence social support for Chinese and Korean immigrants. Therefore, we included other variables that we hypothesized might be associated with social support. Household size may influence social support, since having a larger household might increase the chances that those people in the household offer instrumental and emotional support to one another (Thomas et al., 2021; Yu et al., 2020). Household size was determined by asking respondents how many people lived in their household, including themselves.

We were also interested to determine if factors related to immigration were associated with social support. For example, social support might be lower when immigrants first arrive in the U.S., but with greater length of stay may have increasing levels of social support (Harley and Eskenazi, 2006). Years in the U.S. was calculated by subtracting the year that the respondent reported coming to the U.S. from the year of interview. Ethnic identity may also influence social support. People who closely identify with their Asian identities may be better connected to their Asian immigrant communities, and therefore have strong social support (Lu et al., 2019). On the other hand, people who are more “westernized” may have more social connections with people outside of the Asian immigrant community, and therefore may find greater social support in the U.S. (Lim et al., 2008; Sullivan and Kashubeck-West, 2015). We included a measure of ethnic identity to explore this. This measure of ethnic identity was one item adapted from the Suinn-Lew Asian Self-Identity Acculturation Scale that has been used previously to examine how ethnic identity is associated with health (Lu et al., 2019; Suinn et al., 1992). Respondents were asked to rate themselves as “very Asian,” “mostly Asian,” “bicultural,” “mostly westernized,” or “very westernized.” Few people rated themselves as mostly westernized (five people or 1.25% of the sample) or very westernized (one person or 0.25% of the sample), so we combined them with people identifying as bicultural. The ethnic identity variable used in analysis included three categories: very Asian, mostly Asian, or bicultural/westernized.

Lastly, social support may vary by acculturative stress—defined here as the stressors associated with the immigrant experience and being a racial/ethnic minority in a cultural context that is different from that of one’s country of origin (Smart and Smart, 1995; Yakushko et al., 2008). Acculturative stress was measured with a commonly used scale adopted from the National Latino and Asian American Longitudinal Survey (Gee et al., 2007; Lueck and Wilson, 2010; Singh et al., 2015). The scale included nine items with “yes,” “no,” or “not applicable” responses. The items included questions such as “do you feel guilty for leaving family or friends in your country of origin?” “do you find it hard interacting with others because of difficulties you have with the English language?” and “do you avoid seeking health services due to fear of immigration officials?” Each “yes” response was coded as one, while any other response was coded as zero. The items were summed to create an acculturative stress score with a potential range of zero to nine, with higher scores indicating greater acculturative stress (Cronbach’s alpha: 0.57).

### 2.3. Analysis

We conducted all analyses in Stata version 16 (StataCorp, 2019). First, we examined the descriptive statistics for all the study variables. Then, we conducted a series of multivariable logistic regression models estimating the odds ratios for fair/poor versus good/very good/excellent SRH, predicted on social support. The first logistic regression model included only social support as an independent variable. The second logistic regression model included demographic and socioeconomic characteristics that might confound the association between social support and SRH: age, gender, marital status, Asian subgroup, education, income, employment, and health insurance status. The third model included perceived stress, a potential explanatory factor of the association between social support and SRH. The fourth and final model added perceived distress, another potential explanatory factor of the association between social support and SRH. We assessed models for multicollinearity.

Next, we conducted a mediation analysis to estimate if perceived stress and distress explained the association between social support and SRH. This analysis determined how much of the main association (social support predicting SRH) was attributable to the potential mediators (perceived stress and distress). The mediation analysis was conducted in Stata using the Karlson, Holm, and Breen (KHB) method (Kohler et al., 2011). This method allowed us to decompose the total effect of social support on SRH, the direct (unmediated) effect of social support on SRH, and the indirect (mediated) effect of social support on SRH through perceived stress and distress. KHB is appropriate for estimating these effects with nonlinear probability models such as those calculated using logistic regression. The KHB method also provides us with a summary of the percentage of the total effect of social support on SRH that is due to perceived stress alone, distress alone, and perceived stress and distress together.

We conducted a series of nested multivariable linear regression models to determine which factors are associated with social support for Chinese and Korean immigrants. The first model included demographic characteristics: age, gender, marital status, and Asian subgroup. The second model added socioeconomic variables (education, income, employment), health insurance, and household size. The third model included years in the U.S. The fourth model added ethnic identity. The fifth and final model added acculturative stress.

Additionally, we conducted a sensitivity analysis using Poisson regression models to calculate the incident-rate ratios of fair/poor SRH on social support and covariates. Poisson regression is a commonly used method for calculating incident-rate ratios, which can be interpreted as prevalence ratios when using cross-sectional datasets to predict outcomes that occur in the sample at a prevalence of more than 10% (Coutinho et al., 2008; Tamhane et al., 2016). We also used the KHB method to decompose the total effect, direct effect, and indirect effect of social support on the incident rate ratio of fair/poor SRH using the Poisson models. However, the KHB results that calculate mediation from Poisson regression models are considered experimental at this time (Kohler et al., 2011; Smith, Lacy, and Mayer, 2019).

## 3. Results

Table 1 shows the descriptive statistics for the STOP CRC baseline survey. The average age in the sample was 58 years old. Respondents were slightly more likely to identify as female (52.8%) than male (47.3%) and the majority were married or cohabitating (85.3%). Education varied in the sample, although almost half of the sample were college graduates and above. Income also varied considerably; 15.5% had income of less than $20,000 while 27% had income of $100,000 or more. The majority of the sample were employed full time (57.8%) and had private insurance (60.5%). Average perceived stress score was 15.62 out of a possible range of zero to 40, while average perceived distress score was 3.65 out of a possible range of zero to 10. Average household size was 2.95 people, and average years lived in the U.S. was 23.11. With regards to ethnic identity, 60.5% identified as “very Asian,” 15.5% identified as “mostly Asian,” and 24% identified as “bicultural or westernized.” The average acculturative stress score was 1.64 out of a possible range of zero to nine. Social support on average was 3.74 out of a possible range of one to five. Slightly more than half of the sample (55.8%) rated their health as good/very good/excellent, while the remaining 44.3% rated their health as fair/poor.

Table 2 shows the logistic regression results of the odds of fair/poor SRH on social support and covariates. In Model 1, more social support was associated with improved SRH. For every one-point increase in the social support score, the odds of fair/poor SRH were lower by 43%. When age, gender, marital status, Asian subgroup, education, income, employment, and health insurance status were included in Model 2, the lower odds of fair/poor SRH with higher social support remained. In this model, female participants were 83% more likely than male participants to report fair/poor SRH. Higher levels of income were associated with lower odds of fair/poor health. For example, those with income of $100,000 or more were 69% less likely to report fair/poor SRH than those with income of less than $20,000. None of the other covariates were strongly associated with SRH in Model 2.

In Model 3, perceived stress was added to the logistic regression model. In this model, social support remained associated with SRH, but to a lesser extent. For every one-point increase in the social support score, the odds of fair/poor SRH were lower by 34%. Perceived stress was strongly associated with SRH in Model 3, such that for every one-point increase in the perceived stress score, the odds of fair/poor SRH was higher by 12%. In Model 4, perceived distress was included to the logistic regression. The association between social support and SRH remained but was slightly less robust than in Model 3. For every one-point increase in social support score, the odds of fair/poor SRH were lower by 30%. Perceived distress was associated with SRH, such that every one-point increase in distress was associated with 15% lower odds of fair/poor SRH. Greater perceived stress remained associated with fair/poor SRH in Model 4. Furthermore, male compared to female, graduate/professional school education compared to less than high school education, and greater income compared to lower income were all associated with lower odds of fair/poor SRH in the final model. Age, marital status, Asian subgroup, employment, and health insurance status were not highly associated with SRH in any of the logistic regression models. There was no evidence of multicollinearity in the final model.

Table 3 presents the results of the mediation analysis conducted using the KHB method. First, the table shows the total effect of social support on fair/poor SRH. This is followed by the decomposed direct (i. e. unmediated) effect of social support on fair/poor SRH and indirect (i. e. mediated) effect of social support on fair/poor SRH through perceived stress and distress. All these effects were calculated accounting for the following covariates: age, gender, marital status, Asian subgroup, education, income, employment, and health insurance status. Note that the calculated odds ratios for the total, direct, and indirect effects presented in Table 3 are on the same parameter scale (Kohler et al., 2011). These estimates are therefore not comparable to the odds ratios in Table 2, which presents logistic regression results for nested nonlinear probability models that have different scale parameters depending on the independent variables included in each model. The total effect of social support on fair/poor SRH was an odds ratio of 0.54 (95% CI: 0.41–0.71). There remained a direct effect of social support on fair/poor SRH independent of the potential mediators (OR: 0.70, 95% CI: 0.53–0.94). The odds ratio for the indirect effect of social support on fair/poor SRH through perceived stress and distress was 0.77 (95% CI: 0.67–0.88), indicating that there was a mediated effect with perceived stress and distress explaining some of the association between social support and SRH.

The KHB method summarizes the mediation effect due to perceived stress and distress at the bottom of Table 3. Of the total effect of social support on fair/poor SRH, 24.97% of the effect was due to perceived stress alone. Similarly, 18.01% of the total effect was due to perceived distress alone. Together, perceived stress and distress account for 42.98% of the total effect of social support on fair/poor SRH, accounting for all other covariates.

Table 4 presents the multivariable linear regression estimating social support. In Model 1, age and Asian subgroup were not highly associated with social support. However, female participants had higher levels of social support than male participants, and unmarried participants had lower social support than married participants. Model 2 added socioeconomic, health insurance, and household size variables. Education, income, health insurance, and household size were not highly associated with social support. However, those who were not currently employed had higher levels of social support than those who were employed full time. In Model 3, longer time lived in the U.S. was associated with greater social support, such that for every additional year lived in the U. S., there was an estimated 0.011 higher score of social support. The associations between the other covariates and social support remained similar in Model 3 as in Model 2. In Model 4, ethnic identity was associated with social support. Those who identified as “bicultural/westernized” had higher level of social support as compared to those who identified as “very Asian.” When ethnic identity was added to the linear regression in Model 4, the association between years lived in the U.S. and social support remained in the same direction as in Model 3 but was no longer statistically significant (p > 0.1). Model 5 added acculturative stress to the previous Model 4. A one-point higher acculturative stress score was associated with a 0.12 lower social support score. The association between ethnic identity and social support remained similar in Model 5 as in Model 4. With regards to the other covariates in this final model, age, gender, Asian subgroup, education, income, household size, and years in the U.S. were not greatly associated with social support. Meanwhile, being married compared to being unmarried, being not employed compared to working full time, and having private insurance compared to having Medicare/Medicaid were associated with higher levels of social support.

In sensitivity analysis, we conducted a series of Poisson regression models to calculate prevalence ratios of fair/poor SRH Table Appendix 1). The results were similar to those in Table 2. We also conducted a second KHB analysis using Poisson regression instead of logistic regression Table Appendix 2). These findings were similar to the results in Table 3, but the results using Poisson regression are considered experimental (Kohler et al., 2011).

## 4. Discussion

We sought to test the role of social support on the health of Chinese and Korean immigrants in the U.S. Specifically, we examined whether social support increased the odds of better SRH, and whether this could be partly explained by higher social support lowering perceived stress and distress. Finally, we explored demographic, socioeconomic, and immigration-related factors that are associated with social support for Chinese and Korean immigrants. Our findings indicated that greater social support indeed coincides with better SRH among foreign-born Chinese and Korean Americans, supporting our first hypothesis. Furthermore, perceived stress and distress together explained nearly half of the effect of social support on SRH, supporting our second hypothesis. This was mostly driven by perceived stress, which accounted for one-quarter of the association between social support and SRH. Perceived distress was also a mediator, explaining 18% of the association between social support and SRH.

These findings indicate the importance of social support to the health of Chinese and Korean immigrants. This coincides with other literature showing the associations between social support and SRH for populations broadly (Caetano et al., 2013; Donato et al., 2018; Matud, García, and Fortes, 2019; White et al., 2009) and for Chinese populations in China (Dai et al., 2016; Yang and Jiang, 2020). The current research adds to a growing body of work examining social support mechanisms for the health of Asian American immigrants (Singh et al., 2015; Fang et al., 2020; Lu et al., 2019; Sangalang and Gee, 2012). Furthermore, we contribute to the research on social support and health among Asian American immigrants by demonstrating the mediating roles of perceived stress and distress in the association between social support and SRH. This confirms some of the foundational theoretical work in this area that suggests that social support influences health through psychological processes such as appraisals, emotions, and feelings of control (Cohen and Wills, 1985; Uchino, 2006). Our findings uphold the theory that social support directly and positively affects overall well-being, while also indirectly affecting health by optimizing stress appraisals, which is commonly known as the stress-buffering effect of social support (Cohen and Wills, 1985; Thoits, 2011). The current study expands the applicability of this theoretical perspective beyond the western individualistic cultural context to Chinese and Korean immigrants in the U. S., as researchers have previously pointed out to be an important area of future investigation (Sangalang and Gee, 2012; Thoits, 2011).

Previous studies have demonstrated the stress-buffing effect of social support by examining how social support moderates the effect of a stressor on health for Asian Americans (Fang et al., 2020; Lu et al., 2019; Singh et al., 2015). A moderation analysis may be most apt when examining the role of social support in buffering against the negative effects of a stressor (i.e., stressful event), while a mediation analysis may be more appropriate when examining the role of stress appraisals (i.e., evaluation and coping response to a stressful event) as mechanisms linking social support and health (Szkody and McKinney, 2020). In this study, we suggest that our measure of perceived stress captures stress appraisal, rather than the stressor. By examining the mediating role rather than the moderating role of perceived stress, we are capturing how social support influences stress appraisals, which then influences SRH. This is a contribution to the literature on social support for Chinese and Korean Americans that has not been demonstrated previously.

Having demonstrated the central role of social support in the health of Chinese and Korean immigrants, we explored which sociodemographic factors contributed to social support. Female gender was associated with slightly more social support than male gender, as has been seen in other studies among Chinese immigrants (Fang et al., 2020). However, gender was not the most salient factor in predicting social support, especially after accounting for other covariates. Other previous work has shown little differences in social support by gender among Asian Americans (Sangalang and Gee, 2012). Being married was strongly and positively associated with greater social support for Chinese and Korean immigrants. Past research on social support has demonstrated that marriage can be an important source of emotional and instrumental support (Donnelly et al., 2019; Soulsby and Bennett, 2015; Umberson et al., 2016). This may be especially true for Asian immigrants in the U.S., who may have migrated together as married couples or who married after migration but share a similar immigrant experience. The role of marriage as a source of social support may be even more important for Asian immigrants, who are likely living apart from extended family member networks for longer periods (Vesely et al., 2016). Asian Americans have higher rates of marriage than the U.S. population as a whole, which may indicate the importance of marriage in the Asian immigrant context as a source of valuable social support (Pew Research Center, 2013). Of the socioeconomic factors, only employment was associated with social support: those who were not employed had higher social support than full-time workers. Not currently employed respondents may be retired or may be homemakers, and they possibly have more time to focus on building social support than those who are working (Goodman et al., 2017).

Regarding immigration factors associated with social support, years lived in the U.S. appeared to be positively associated with greater social support (Ro, 2014). However, this association was not as strong as ethnic identity. Ethnic identity explained more of the variance in social support than years in the U. S., with people identifying as “bicultural” or “westernized” experiencing higher levels of social support than those who identified themselves as “very Asian.” This finding seems to contradict prior work that shows that among Chinese immigrants, upholding Chinese cultural values was associated with greater perceived availability of social support (Lee et al., 2012). It is possible that people who are more acculturated and able to navigate both Asian and western contexts have higher levels of social support from wider social networks (Lim et al., 2008), which may come in the form of instrumental or informational support (Sullivan and Kashubeck-West, 2015). Future work should consider examining the types of social support that Asian immigrants receive from their co-ethnic compared to their non-Asian social networks, to determine whether acculturation is consistently associated with greater social support. Greater acculturative stress was associated with lower levels of social support in this study. This finding coincides with previous literature showing that social support is negatively associated with acculturative stress (Fang et al., 2020; Sangalang and Gee, 2012; Singh et al., 2015). Acculturative stress explained the greatest amount of variance in social support compared to the other immigration factors. However, the measure of acculturative stress had low reliability, so findings should be interpreted with some caution. Although the cross-sectional analysis limits our ability to determine directionality, future longitudinal work can consider the possible bidirectional relationship between acculturative stress and social support. It is possible that experiencing acculturative stress can interfere with building social support, and it is also possible that low levels of social support can contribute to greater acculturative stress. In sum, our third hypothesis that factors related to immigration were associated with social support was confirmed. Ethnic identity and acculturative stress showed strong associations with social support among Chinese and Korean immigrants.

### 4.1. Limitations

The cross-sectional nature of the dataset is one of the main limitations of this research. In this study, we attempted to conduct a mediation analysis to understand how social support contributes to lowering psychological stress, which leads to better SRH. However, we recognize that in a cross-sectional analysis, we cannot determine temporal ordering of the variables. Therefore, we cannot rule out the possibility that the associations may be in the other direction—for example, poor SRH could increase stress, which then lowers social support. Although a majority of longitudinal studies find exposure to stress results in worsened health over time (Huang et al., 2021; Moskowitz et al., 2013), research has also shown that poor health could itself be a major stressor, preceding more experiences of stress and distress (Compas et al., 2012; Currie, 2009). Some studies also report that people who experience high levels of stress and distress have lessened social support as a result (Platt et al., 2016; Thompson and Goodvin., 2016). Therefore, it is possible that poor SRH can decrease people’s availability of social support via increased stress (Cohen, 1992; Compas et al., 2012). Nevertheless, this paper builds upon theories and research of how social support is related to health, with findings that are consistent with what prior literature has shown (Lu et al., 2019; Thoits, 2011; Uchino, 2006).

Although this is a unique sample of Chinese and Korean immigrants that allows us to examine in-depth how experiences related to immigration are associated with social support and health, these findings may not be generalizable to all Asian immigrants, nor to U.S.-born Asian Americans. Future work should examine whether similar findings are true of other Asian ethnic subpopulations in other geographic locations outside the Baltimore-Washington DC Metropolitan Area. It is also worthwhile to examine how associations between social support, psychological stress, health, and cultural factors differ or remain the same for U.S.-born Asian American groups in the second and subsequent generations.

## 5. Conclusion

In conclusion, this paper demonstrates the centrality of the role of social support affecting the health of Chinese and Korean immigrants in the U.S. through the mechanisms of psychological stress and distress. Furthermore, social support among Chinese and Korean immigrants is highly influenced by factors related to the immigrant experience, including acculturation and acculturative stress (Lee and Ciftci, 2014; Sangalang and Gee, 2012; Umaña-Taylor, 2011). These findings open the door for future hypothesis testing on the stress-buffering effect of social support on multiple health outcomes among Asian American groups. This research also sets groundwork for future health interventions to consider strengthening social support particularly among immigrant groups that may lack support, to lessen psychological stress related to poor health outcomes.

## Figures and Tables

**Table 1:** Descriptive statistics, STOP CRC baseline data 2018–2020. N = 400.

Variables	Mean (SE) or Freq (%)
Age	58.39 (6.36)
Gender	
Male	189 (47.3%)
Female	211 (52.8%)
Marital status	
Married/Cohabitating	341 (85.3%)
Not currently married	59 (14.8%)
Asian Subgroup	
Korean	200 (50%)
Chinese	200 (50%)
Education	
Less than high school	43 (10.8%)
High school grad/GED	91 (22.8%)
Some college/vocational school	68 (17%)
College graduate	101 (25.3%)
Attended graduate/professional school	97 (24.3%)
Income	
Less than $20,000	62 (15.5%)
$20,000-$39,000	64 (16%)
$40,000-$59,000	85 (21.3%)
$60,000-$79,000	49 (12.3%)
$80,000-$99,000	32 (8%)
$100,000 or more	108 (27%)
Employment	
Full time	231 (57.8%)
Part time	84 (21%)
Not employed	85 (21.3%)
Health Insurance	
Private	242 (60.5%)
Medicare/Medicaid	74 (18.5%)
None	84 (21%)
Perceived stress (30 day, range: 0–40)	15.62 (4.32)
Perceived distress (past week, range: 0–10)	3.65 (2.42)
Household size	2.95 (1.23)
Years in the U.S.	23.11 (10.28)
Ethnic Identity	
Very Asian	242 (60.5%)
Mosdy Asian	62 (15.5%)
Bicultural/Westernized	96 (24%)
Acculturative Stress (range: 0–9)	1.64 (1.55)
Social Support (range: 1–5)	3.74 (0.89)
Self-Rated Health	
Good/Very good/Excellent	223 (55.8%)
Fair/Poor	177 (44.3%)

**Table 2:** Logistic regression of fair/poor self-rated health on social support and covariates, STOP CRC baseline data 2018–2020. N = 400.

Variables	OR (95% CI)			
	Model 1	Model 2	Model 3	Model 4
Social support	0.57*** (0.45–0.73)	0.56*** (0.43–0.73)	0.66** (0.50–0.88)	0.70* (0.53–0.94)
Age		1.00 (0.96–1.04)	1.01 (0.96–1.05)	1.01 (0.97–1.05)
Female (ref = male)		1.83* (1.13–2.96)	1.74* (1.07–2.85)	1.72* (1.04–2.82)
Unmarried (ref = married)		0.88 (0.46–1.67)	0.89 (0.46–1.72)	0.88 (0.45–1.71)
Chinese (ref = Korean)		0.86 (0.52–1.42)	1.00 (0.60–1.68)	0.99 (0.59–1.67)
Education (ref = less than HS)				
HS graduate/GED		1.28 (0.58–2.79)	1.38 (0.62–3.07)	1.37 (0.61–3.06)
Some college		1.16 (0.51–2.68)	1.31 (0.56–3.06)	1.21 (0.51–2.85)
College graduate		1.08 (0.49–2.38)	1.10 (0.49–2.46)	0.99 (0.44–2.23)
Graduate/professional school		0.42† (0.17–1.06)	0.44† (0.17–1.12)	0.36* (0.14–0.94)
Income (ref = less than $20K)				
$20-$39K		0.75 (0.34–1.67)	0.67 (0.30–1.53)	0.64 (0.28–1.47)
$40–59K		0.46† (0.20–1.02)	0.51 (0.22–1.16)	0.46† (0.20–1.08)
$60–79K		0.34* (0.14–0.84)	0.36* (0.14–0.92)	0.32* (0.13–0.82)
$80–99K		0.26* (0.092–0.74)	0.26* (0.092–0.76)	0.24** (0.084–0.71)
$100K+		0.31* (0.13–0.76)	0.39* (0.15–0.99)	0.37* (0.15–0.95)
Employment (ref = full time)				
Part time		1.23 (0.68–2.24)	1.40 (0.75–2.59)	1.47 (0.79–2.75)
Not employed		1.18 (0.62–2.22)	1.14 (0.59–2.18)	1.17 (0.60–2.25)
Health insurance (ref = private)				
Medicare/Medicaid		0.75 (0.37–1.50)	0.75 (0.37–1.52)	0.73 (0.36–1.51)
None		1.21 (0.67–2.18)	1.20 (0.66–2.18)	1.22 (0.67–2.22)
Perceived stress (30 days)			1.12*** (1.06–1.19)	1.09** (1.02–1.17)
Perceived distress (week)				1.15** (1.04–1.28)
Constant	6.27*** (2.52–15.6)	12.0† (0.65–222)	0.55 (0.018–16.5)	0.41 (0.013–12.7)

† p < 0.1

* p < 0.05

** p < 0.01

*** p < 0.001, OR = odds ratio, HS = high school, GED = general education degree.

**Table 3:** Summary of the decomposition of the total effect of social support on fair/poor self-rated health into the direct unmediated effect and the indirect mediated effect through perceived stress and distress, estimated using logistic regression, STOP CRC baseline data 2018–2020. N = 400.

Decomposition of effects		OR (95% CI)	*p*-Value
Total effect of social support on fair/poor self-rated health		0.54 (0.41–0.71)	0.000
Direct (unmediated) effect of social support on fair/poor self-rated health		0.70 (0.53–0.94)	0.016
Indirect (mediated) effect of social support on fair/poor self-rated health through perceived stress and distress		0.77 (0.67–0.88)	0.000
Summary of mediation	%		
Percent of total effect due to perceived stress alone	24.97%		
Percent of total effect due to perceived distress alone	18.01%		
Percent of total effect due to both perceived stress and distress	42.98%		

Note: All calculated effects account for age, gender, marital status, Asian subgroup, education, income, employment, and health insurance status.

**Table 4:** Multivariable linear regression of social support on covariates, STOP CRC baseline data 2018–2020. N = 400.

	Coef. (SE)				
VARIABLES	Model 1	Model 2	Model 3	Model 4	Model 5
Age	0.0046 (0.0072)	0.0055 (0.0087)	0.0023 (0.0088)	0.0025 (0.0088)	0.0047 (0.0086)
Female (ref = male)	0.20* (0.088)	0.16† (0.095)	0.17† (0.095)	0.16† (0.094)	0.15 (0.092)
Unmarried (ref = married)	−0.46*** (0.12)	−0.46*** (0.13)	−0.48*** (0.13)	−0.51*** (0.13)	−0.47*** (0.13)
Chinese (ref = Korean)	0.075 (0.091)	−0.018 (0.10)	0.043 (0.10)	−0.14 (0.12)	−0.17 (0.12)
Education (ref = less than HS)					
HS graduate/GED		−0.10 (0.16)	−0.12 (0.16)	−0.15 (0.16)	−0.15 (0.16)
Some college		0.093 (0.17)	0.058 (0.17)	−0.019 (0.17)	−0.056 (0.17)
College graduate		0.049 (0.17)	0.051 (0.17)	−0.00054 (0.16)	−0.036 (0.16)
Graduate/professional school		0.15 (0.18)	0.13 (0.18)	0.028 (0.18)	−0.017 (0.18)
Income (ref = less than $20K)					
$20-$39K		0.053 (0.16)	0.053 (0.16)	0.0024 (0.16)	0.028 (0.16)
$40–59K		−0.065 (0.17)	−0.095 (0.17)	−0.17 (0.17)	−0.14 (0.16)
$60–79K		0.028 (0.18)	−0.0051 (0.18)	−0.072 (0.18)	−0.067 (0.18)
$80–99K		0.098 (0.21)	0.081 (0.21)	0.018 (0.20)	−0.013 (0.20)
$100K+		0.18 (0.18)	0.11 (0.18)	0.047 (0.18)	0.043 (0.18)
Employment (ref = full time)					
Part time		0.16 (0.12)	0.18 (0.12)	0.21† (0.12)	0.19 (0.12)
Not employed		0.27* (0.13)	0.32* (0.13)	0.34** (0.13)	0.33** (0.13)
Health insurance (ref = private)					
Medicare/Medicaid		−0.17 (0.14)	−0.22 (0.14)	−0.26† (0.14)	−0.28* (0.14)
None		−0.18 (0.12)	−0.16 (0.12)	−0.19 (0.12)	−0.17 (0.12)
Household size		−0.027 (0.038)	−0.027 (0.038)	−0.019 (0.038)	−0.025 (0.037)
Years in the U.S.			0.011* (0.0050)	0.0075 (0.0050)	0.0027 (0.0050)
Ethnic identity (ref = very Asian)					
Mostly Asian				0.25† (0.15)	0.20 (0.14)
Bicultural/Westernized				0.41*** (0.12)	0.35** (0.12)
Acculturative stress					−0.12*** (0.029)
Constant	3.40*** (0.44)	3.37*** (0.60)	3.31*** (0.60)	3.43*** (0.59)	3.70*** (0.58)
R-squared	0.041	0.087	0.098	0.124	0.165

† p < 0.1

* p < 0.05

** p < 0.01

*** p < 0.001, HS = high school, GED = general education degree

## Appendix

**Appendix 1:** Poisson regression of fair/poor self-rated health on social support and covariates, STOP CRC baseline data 2018–2020. N = 400

	IRR (95% CI)			
Variables	Model 1	Model 2	Model 3	Model 4
Social support	0.76*** (0.68–0.85)	0 78*** (0.70–0.87)	0.85** (0.76–0.96)	0.88* (0.78–0.99)
Age		1.00 (0.98–1.02)	1.00 (0.98–1.02)	1.00 (0.98–1.02)
Female (ref = male)		1.31* (1.05–1.65)	1.30* (1.04–1.62)	1.28* (1.03–1.60)
Unmarried (ref = married)		0.95 (0.71–1.26)	0.95 (0.72–1.26)	0.95 (0.72–1.25)
Chinese (ref = Korean)		0.92 (0.73–1.17)	0.99 (0.79–1.25)	0.98 (0.78–1.24)
Education (ref = less than HS)				
HS graduate/GED		1.08 (0.79–1.49)	1.13 (0.83–1.55)	1.12 (0.81–1.54)
Some college		1.05 (0.74–1.51)	1.13 (0.79–1.62)	1.08 (0.75–1.54)
College graduate		1.02 (0.72–1.45)	1.03 (0.73–1.46)	0.97 (0.68–1.38)
Graduate/professional school		0.57* (0.34–0.95)	0.59* (0.36–0.98)	0.54* (0.33–0.89)
Income (ref = less than $20K)				
$20-$39K		0.91 (0.67–1.23)	0.89 (0.66–1.21)	0.87 (0.64–1.17)
$40–59K		0.73# (0.52–1.03)	0.79 (0.57–1.10)	0.75# (0.54–1.04)
$60–79K		0.64* (0.41–0.99)	0.68# (0.44–1.04)	0.64* (0.42–0.98)
$80–99K		0.53* (0.32–0.88)	0.56* (0.33–0.94)	0.54* (0.32–0.89)
$100K+		0.55** (0.35–0.86)	0.63* (0.40–1.00)	0.62* (0.39–0.98)
Employment (ref = full time)				
Part time		1.10 (0.84–1.42)	1.14 (0.88–1.46)	1.17 (0.91–1.51)
Not employed		1.11 (0.83–1.48)	1.07 (0.81–1.42)	1.10 (0.83–1.45)
Health insurance (ref = private)				
Medicare/Medicaid		0.85 (0.62–1.15)	0.86 (0.64–1.17)	0.84 (0.62–1.13)
None		1.06 (0.83–1.37)	1.07 (0.83–1.37)	1.08 (0.84–1.39)
Perceived stress (30 days)			1.06*** (1.03–1.09)	1.05** (1.01–1.08)
Distress (week)				1.06** (1.02–1.11)
Constant	1.19 (0.83–1.72)	1.34 (0.36–4.96)	0.28 (0.059–1.29)	0.25# (0.051–1.19)

† p < 0.1

* p < 0.05

** p < 0.01

*** p < 0.001, IRR = incidence rate ratio, HS = high school, GED = general education degree.

**Appendix 2:** Summary of the decomposition of the total effect of social support on fair/poor self-rated health into the direct unmediated effect and the indirect mediated effect through perceived stress and distress, estimated using Poisson regression, STOP CRC baseline data 2018–2020. N = 400

Decomposition of effects	IRR (95% CI)	*p*-Value
Total effect of social support on fair/poor self-rated health	0.77 (0.66–0.91)	0.002
Direct (unmediated) effect of social support on fair/poor self-rated health	0.88 (0.74–1.05)	0.144
Indirect (mediated) effect of social support on fair/poor self-rated health through perceived stress and distress	0.81 (0.81–0.96)	0.003
Summary of mediation	%	
Percent of total effect due to perceived stress alone	30.57%	
Percent of total effect due to distress alone	18.64%	
Percent of total effect due to both perceived stress and distress	49.21%	

Note: All calculated effects account for age, gender, marital status, Asian subgroup, education, income, employment, and health insurance status. The KHB method used to decompose these effects in Poisson regression are considered experimental.
